# Bone Tissue Regeneration by Collagen Scaffolds with Different Calcium Phosphate Coatings: Amorphous Calcium Phosphate and Low-Crystalline Apatite

**DOI:** 10.3390/ma14195860

**Published:** 2021-10-07

**Authors:** Syama Santhakumar, Ayako Oyane, Maki Nakamura, Yuto Yoshino, Mohammed Katib Alruwaili, Hirofumi Miyaji

**Affiliations:** 1 Nanomaterials Research Institute, National Institute of Advanced Industrial Science and Technology (AIST), Central 5, 1-1-1 Higashi, Tsukuba, Ibaraki 305-8565, Japan; ma-ki-nakamura@aist.go.jp; 2 Department of Periodontology and Endodontology, Faculty of Dental Medicine, Hokkaido University, N13W7, Kita-ku, Sapporo, Hokkaido 060-8586, Japan; yutoy0526@den.hokudai.ac.jp (Y.Y.); mohammed.katib@jodent.org (M.K.A.)

**Keywords:** apatite, amorphous calcium phosphate (ACP), bone tissue engineering, scaffolds, mineralization

## Abstract

Surface-mineralized collagen sponges have attracted much attention as scaffolds for bone tissue engineering. Recently, we developed amorphous calcium phosphate (ACP) and low-crystalline apatite coating processes on collagen sponges. In the present study, we applied these coating processes to granular collagen sponges (referred to as Col) to compare the bone tissue regeneration capabilities of ACP-coated and apatite-coated Col (referred to as Col-ACP and Col-Ap, respectively) using a rat cranial bone defect model. According to micro-CT and histological analyses, Col-Ap enhanced bone tissue regeneration compared to Col, whereas Col-ACP did not. These results not only demonstrated the superior bone tissue regeneration capability of Col-Ap, but also indicated limitations of the in vitro simulated body fluid (SBF) test used in our previous study. Despite the apatite-forming ability of Col-ACP in SBF, it was ineffective in improving bone tissue regeneration in vivo, unlike Col-Ap, most likely due to the quick resorption of the ACP coating in the defect site. The present results clarified the importance of the coating stability in vivo and revealed that the low-crystalline apatite coating was more beneficial than the ACP coating in the fabrication of surface-mineralized collagen sponges for use as bone tissue engineering scaffolds.

## 1. Introduction

Biodegradable scaffolds with a three-dimensional (3D) porous structure play an important role in bone tissue engineering. They support cell adhesion, growth, and osteogenic differentiation; facilitate the formation of blood vessels; and finally, resorb while being replaced with the newly formed bone. In human bone, collagen fibrils mineralized with nonstoichiometric low-crystalline apatite constitute the basic building blocks [1]. Hence, biocompatible and bioresorbable collagen sponges coated with low-crystalline apatite have attracted attention as scaffolds for bone tissue engineering owing to their similarity to human bone. Several reports have shown that such apatite-coated collagen sponges enhance bone tissue regeneration compared to bare sponges without coating [2,3,4]. However, apatite is formed via the formation of thermodynamically unstable precursor phases under physiological conditions [5,6,7]. In bone biomineralization, a non-crystalline phase of calcium phosphate is concentrated within the intracellular vesicles of osteoblasts [8,9], and later, infiltrates the collagen fibrils where it crystallizes to form low-crystalline apatite [10,11]. Because of the critical role of amorphous calcium phosphate (ACP) in biomineralization, ACP-coated collagen sponges have also emerged as potential scaffolds for bone tissue engineering [12,13]. However, there have been few comparative analyses of apatite- and ACP-coated collagen sponges for use as bone tissue engineering scaffolds.

Recently, we fabricated two types of mineralized porous collagen scaffolds for bone tissue engineering: collagen rectangular blocks coated with low-crystalline apatite [14] and collagen cubic granules coated with ACP nanoparticles [15]. The former scaffold with an apatite coating enhances the adhesion of pre-osteoblastic MC3T3-E1 cells in vitro and enhances cell-ingrowth within its porous body in rat subcutaneous tissue compared to an uncoated scaffold [14]. Moreover, the apatite-coated scaffold significantly promotes cranial bone formation in a rat skull defect model and alveolar bone regeneration in a dog premolar furcation defect model compared to the uncoated scaffold [16]. The latter scaffold with an ACP coating forms an apatite layer on its surface in a simulated body fluid (SBF) test [17], suggesting its apatite-forming ability in vivo and subsequent osteoconductive abilities [15]; however, its bone tissue regeneration capability remains to be elucidated.

Here, we compared the effects of ACP and apatite coatings on a porous collagen scaffold for bone tissue regeneration. In the previous studies described above, the dimensions of the two mineralized collagen scaffolds were different: cubic granules (1 mm × 1 mm × 1 mm) were used for ACP coating [15], whereas larger rectangular blocks (6 mm × 6 mm × 3 mm) were used for apatite coating [14,16]. In the present study, to achieve our aim, both ACP and apatite coatings were prepared on collagen scaffolds of the same size, i.e., cubic granules of a porous collagen sponge (referred to as Col). The ACP-coated and apatite-coated Col (referred to as Col-ACP and Col-Ap, respectively) and uncoated Col as a control were implanted in rat cranial bone defects to compare their bone tissue regeneration capabilities and their potential as bone tissue engineering scaffolds.

## 2. Materials and Methods

### 2.1. Preparation of Scaffolds

The three types of scaffold (Col, Col-ACP, and Col-Ap) were prepared. Cubic granules of Col with a dimension of 1 mm × 1 mm × 1 mm were prepared by cutting a porous sponge sheet of calf atelocollagen (Terudermis, Olympus Terumo Biomaterials Corp., Tokyo, Japan). Col-ACP was fabricated from Col by a temperature-controlled ACP coating process following our previously reported method [15]. Col-Ap was fabricated from Col by a plasma- and precursor-assisted biomimetic process using the same method as described earlier [14], except that 4 mg granules of Col were treated here, instead of three rectangular collagen blocks with dimensions of 6 mm × 6 mm × 3 mm.

### 2.2. Physicochemical Analyses

The scaffolds prepared in the preceding section were analyzed using a field emission scanning electron microscope (SEM; S-4800, Hitachi High-Tech Corporation, Tokyo, Japan) equipped with an energy dispersive X-ray (EDX) spectrometer (EMAX x-act, HORIBA, Ltd., Kyoto, Japan), an X-ray diffraction (XRD) instrument (M18X, MacScience, Tokyo, Japan) with CuKα radiation (λ = 0.154178 nm), and a Fourier transform infrared (FT-IR) spectrometer (FT/IR-4700, JASCO Corporation, Japan) equipped with an attenuated total reflection (ATR) accessory and a monolithic diamond crystal. Prior to the SEM and EDX analyses, the scaffolds were sputter-coated with gold. The XRD measurements were performed at 40 kV and 200 mA with the thin-film mode at an incidence angle of 1°, a 2θ step width of 0.05°, and a counting time of 6 s per step. Cross-sectional ultrathin specimens were prepared from Col-ACP by a conventional resin embedding method and analyzed using an analytical transmission electron microscope (TEM; Tecnai Osiris, FEI, Hillsboro, OR, USA) operated at 200 kV. The crystalline structure of the specimens was examined by selected area electron diffraction (SAED) analyses.

### 2.3. In Vivo Study

In vivo experiments using rats were carried out in accordance with the institutional animal use and care regulations of Hokkaido University and approved by the Animal Research Committee of Hokkaido University (approval number: 19–58). Surgical procedures were performed under general anesthesia by intraperitoneal injection of medetomidine hydrochloride (0.15 µg/kg, Domitor, Nippon Zenyaku Kogyo Co., Ltd., Koriyama, Japan), Midazolam (2.0 µg/kg, Dormicum, Astellas Pharma Inc., Tokyo, Japan), butorphanol tartrate (2.5 µg/kg, Vetorphale, Meiji Seika Pharma Co., Ltd., Tokyo, Japan), and local injection of 2% lidocaine hydrochloride with 1:80,000 epinephrine (Xylocaine Cartridge for Dental Use, Dentsply Sirona K.K., Tokyo, Japan). The three types of scaffolds (Col, Col-ACP, Col-Ap; 4 mg granules per defect) were implanted in defects (5 mm in diameter) created on the cranial bones of Wistar rats (10-week-old, male, 190–210 g) (*n* = 6). After surgery, gentamicin sulfate ointment (GENTACIN Ointment 0.1%, TAKATA Pharmaceutical Co., Ltd. Saitama, Japan) was applied to the wound.

At two weeks post-surgery, rats were euthanized with an overdose of sodium pentobarbital (2.0 mL/kg, Somnopentyl; Kyoritsu Seiyaku Corp., Tokyo, Japan). The cranial bones were extracted and assessed by an X-ray micro-computed tomography (micro-CT) scanner (Latheta LCT-200, Hitachi, Ltd., Tokyo, Japan). From the captured micro-CT images, the radiolucent (non-calcified) area of the cranial bone and the volume of the newly formed bone were measured by image analysis software (ImageJ 1.41; National Institutes of Health, Bethesda, MD, USA). In histological analyses, the extracted cranial bones were fixed in 10% buffered formalin, decalcified with Plank–Rychlo’s solution, and embedded in paraffin. After thin slicing, the tissue sections were stained with Masson’s trichrome and observed using a light microscope (NanoZoomer S210 C13239-01, Hamamatsu Photonics K.K., Hamamatsu, Japan). The area and height of the newly formed bone were measured by image analysis software (ImageJ 1.41). Statistical differences were analyzed by one-way ANOVA with Tukey’s HSD post hoc test (SPSS 11.0, IBM Corporation, Armonk, NY, USA), and differences with *p* < 0.05 were considered statistically significant.

## 3. Results and Discussion

**Figure 1:**
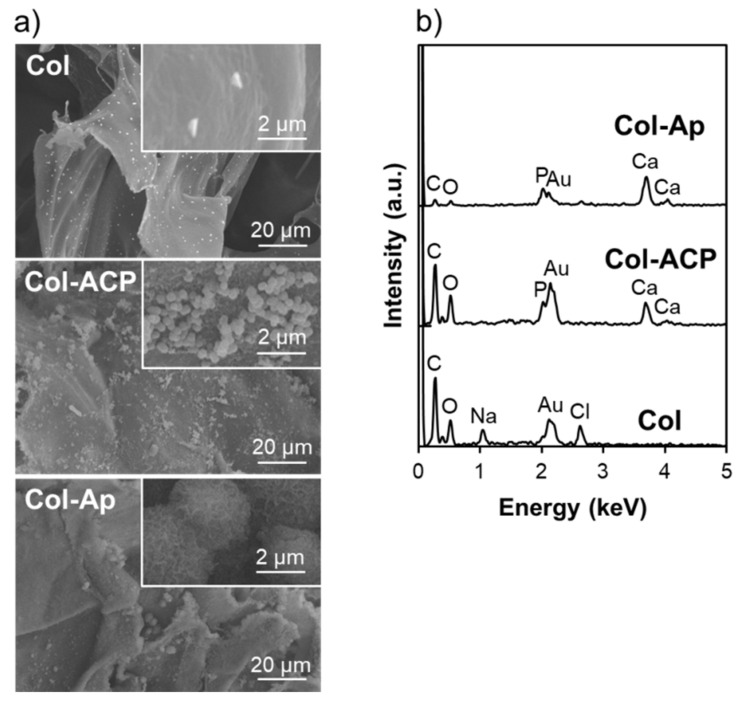
(**a**) SEM images; (**b**) EDX spectra of Col, Col-ACP, and Col-Ap surfaces. Insets in (**a**) show magnified images.

**Figure 2:**
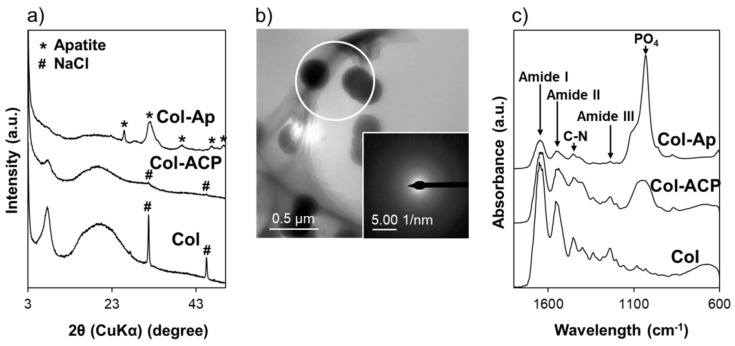
(**a**) XRD patterns of Col, Col-ACP, and Col-Ap surfaces; (**b**) TEM image and SAED pattern of a cross-section of Col-ACP; (**c**) FT-IR spectra of Col, Col-ACP, and Col-Ap surfaces.

The physicochemical analyses revealed successful coating of ACP and low-crystalline apatite on Col-ACP and Col-Ap, respectively. As shown in the SEM images (Figure 1a), spherical nanoparticles and a nanostructured layer appeared on the Col-ACP and Col-Ap surfaces, respectively. Both the spherical nanoparticles and nanostructured layer consisted of calcium phosphate, as indicated by the Ca and P peaks in the EDX spectra of Col-ACP and Col-Ap (Figure 1b). In their XRD patterns, no diffraction peaks were detected for Col-ACP, whereas peaks ascribed to low-crystalline apatite were detected for Col-Ap (Figure 2a). The amorphous nature of the calcium phosphate nanoparticles on Col-ACP was evident from the SAED pattern (Figure 2b). In the FT-IR spectra, both Col-ACP and Col-Ap showed a peak at ~1025 cm^−1^, representing a phosphate group which was absent in Col (Figure 2c). These analytical results are consistent with our previous reports [14,15] and proved that the plasma- and precursor-assisted biomimetic process, which was developed for apatite coating on rectangular collagen blocks [14], was also applicable to the smaller granules of Col.

**Figure 3:**
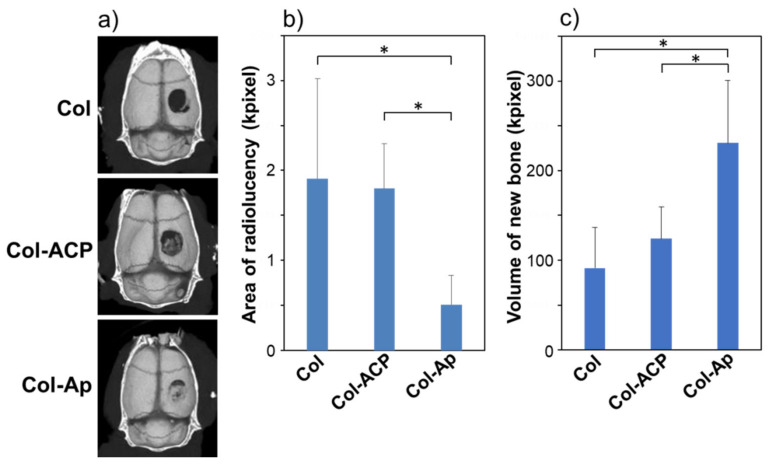
(**a**) Micro-CT images of rat cranial bone two weeks after implantation of Col, Col-ACP, and Col-Ap; (**b**) Radiolucent area (corresponding to the area of the remaining bone defect) and (**c**) volume of new bone from quantitative analyses of the micro-CT images (mean + standard deviation, *n* = 6, * *p* < 0.05).

Col-Ap significantly promoted bone regeneration in rat cranial bone defects compared to Col, although Col-ACP did not. Figure 3a shows representative micro-CT images of the cranial bones implanted with Col, Col-ACP, and Col-Ap. In the Col group, a nearly circular radiolucent (black) area (recognized as a non-calcified area) was clearly observed, indicating that new bone formation hardly occurred in the defect. In the Col-ACP group, a slightly radiopaque (dark gray) area partly appeared in the bone defect, implying its partial repair by the newly formed bone tissue. In the Col-Ap group, a brighter (light gray) radiopaque area spread widely over the bone defect and the residual radiolucent (black) area was very small, suggesting that the bone defect was almost fully repaired by the newly formed bone tissue. The quantitative analysis showed that the radiolucent area in the Col-Ap group was significantly smaller than that in the Col and Col-ACP groups (Figure 3b). Similarly, the volume of the new bone in the Col-Ap group was significantly higher compared to that in the Col and Col-ACP groups (Figure 3c).

**Figure 4:**
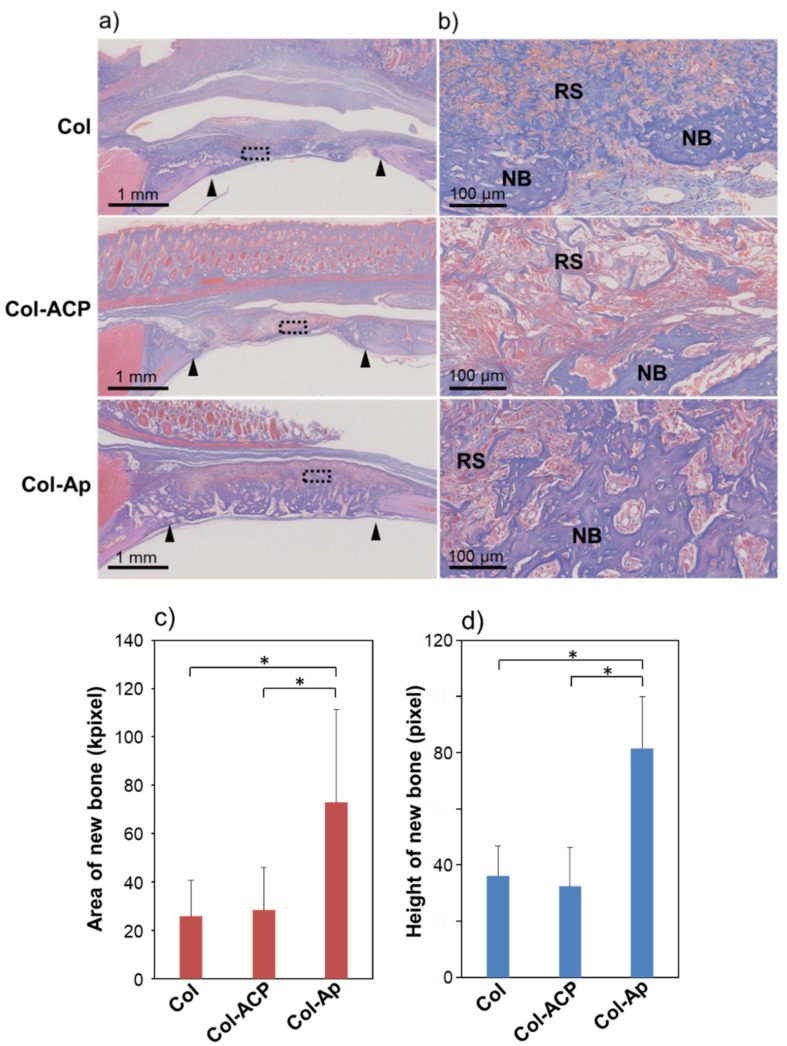
(**a**) Optical microscopic images of the histological sections of the rat cranial bone two weeks after implantation of Col (top row), Col-ACP (middle row), and Col-Ap (bottom row); (**b**) Magnified images of the square region (dashed line squares) in (**a**). (**c**) Area and (**d**) height of the newly formed bone tissue from quantitative analyses of the histological sections (mean + standard deviation, *n* = 6, * *p* < 0.05). Black arrowheads in (**a**) indicate the border of the initially created bone defect. NB and RS in (**b**) indicate newly formed bone tissue and residual scaffold, respectively.

There was no significant difference in the radiolucent area between the Col and Col-ACP groups (*p* > 0.05). These micro-CT results were further supported by the histological observations. In the histological images (Figure 4a,b), the newly formed bone tissue (denoted by NB) was apparent in all groups. Around the newly formed bone tissue, the residual scaffolds (denoted by RS) along with fibroblast-like and macrophage-like giant cells were observed. The area of the newly formed bone tissue in the Col-Ap group was apparently larger than that in the Col and Col-ACP groups, suggesting enhanced bone regeneration by Col-Ap. However, the area of the residual scaffold in the Col-Ap group was apparently smaller than that in the Col and Col-ACP groups, indicating the faster resorption of Col-Ap. Severe inflammatory reactions were not noticed in any group. The histomorphometric analysis clarified that the area and height of the newly formed bone tissue in the Col-Ap group were significantly greater compared to those in the Col and Col-ACP groups (Figure 4c,d). Again, there was no significant difference between the Col and Col-ACP groups (*p* > 0.05). These results reconfirmed the beneficial effect of a low-crystalline apatite coating on Col for bone tissue regeneration. An osteogenic effect by a low-crystalline apatite coating has been reported for similar collagen scaffolds [2,3,4], including for the larger-sized rectangular blocks [16].

Despite its apatite-forming ability in SBF [15], Col-ACP induced different biological responses from Col-Ap; it did not enhance bone regeneration compared to Col in the rat cranial bone defect model (Figure 3 and Figure 4). That is, the present ACP coating on Col had no beneficial effect on bone tissue regeneration apart from the apatite coating, although there are several reports demonstrating the osteogenic effect of ACP [18,19]. Inferior osteogenic effects of ACP coatings compared to apatite coatings have been reported for other coating processes [20,21]. We consider that the ACP nanoparticles on Col-ACP were insufficient in their amount and stability, thereby resorbing in the bone defect before inducing apatite layer formation. A similar phenomenon has been reported for some calcium salts with relatively high solubility, such as calcium sulphate hemihydrate [22] and dicalcium phosphate dihydrate [23]; these materials show poor osteogenic properties, most likely due to fast resorption in vivo (bone defects are repaired only partially by immature bone), despite their apatite-forming ability in SBF [24]. The most probable cause for the discrepancy between the SBF test result [15] and the present in vivo result is the acidified environment resulting from postoperative inflammation reactions [25] and/or acid secretion by osteoclasts [26] in the vicinity of the scaffold. It is known that ACP transforms into apatite under a normal physiological environment, because body fluid (SBF as well) is supersaturated with respect to ACP, apatite, and several other calcium phosphate phases, among which apatite is the most stable and insoluble phase [27]. However, a decrease in environmental pH from the physiological pH (~7.4) significantly increases the solubility of these calcium phosphates [28], which may lead to the dissolution and resorption of the relatively unstable ACP phase. The effects of body fluid circulation might also be involved in the dissolution and resorption of the ACP nanoparticles by facilitating the diffusion of ions and clusters in the amorphous-to-crystalline transformation process through dissolution-reprecipitation reactions [29]. Note that the present results were obtained from rat cranial bone defects. The rate of bone regeneration varies with animal species and the region of bone defects. The flat cranial bone defects are regenerated through the intramembranous ossification process, which is different from longer bone regeneration through the endochondral ossification process. There are reports available on the faster regeneration of longer bone defects compared to that of flat cranial bone defects [30,31]. Hence, these scaffolds might induce different biological responses in different bone defects of different animal species, which are issues for future studies.

## 4. Conclusions

The low-crystalline apatite coating on Col (Col-Ap) was more effective in improving bone tissue regeneration in rat cranial bone defects than the ACP coating (Col-ACP). The stability of the mineralized surface coating is an important factor in determining the bone tissue regeneration capability of the collagen scaffold.

## Data Availability

All data from this study are included within the article.

## References

[1] Jee W.S.S., Weiss L. (1983). The skeletal tissue. Histology, Cell and Tissue Biology.

[2] Fukui N., Sato T., Kuboki Y., Aoki H. (2008). Bone tissue reaction of nano-hydroxyapatite/collagen composite at the early stage of implantation. Biomed. Mater. Eng..

[3] Yang H.S., La W.G., Park J., Kim C.S., Im G.I., Kim B.S. (2012). Efficient bone regeneration induced by bone morphogenetic protein-2 released from apatite-coated collagen scaffolds. J. Biomater. Sci. Polym. Ed..

[4] Liu S., Sun Y., Fu Y., Chang D., Fu C., Wang G., Liu Y., Tay F.R., Zhou Y. (2016). Bioinspired collagen-apatite nanocomposites for bone regeneration. J. Endod..

[5] Meyer J.L., Eanes E.D. (1978). A thermodynamic analysis of the amorphous to crystalline calcium phosphate transformation. Calcif. Tiss. Res..

[6] Eanes E.D., Amjad Z. (1998). Amorphous calcium phosphate: Thermodynamic and kinetic considerations. Calcium Phosphates in Biological and Industrial Systems.

[7] Mahamid J., Sharir A., Addadi L., Weiner S. (2008). Amorphous calcium phosphate is a major component of the forming fin bones of zebrafish: Indications for an amorphous precursor phase. Proc. Natl. Acad. Sci. USA.

[8] Mahamid J., Sharir A., Gur D., Zelzer E., Addadi L., Weiner S. (2011). Bone mineralization proceeds through intracellular calcium phosphate loaded vesicles: A cryo-electron microscopy study. J. Struct. Biol..

[9] Boonrungsiman S., Gentleman E., Carzaniga R., Evans N.D., McComb D.W., Porter A.E., Stevens M.M. (2012). The role of intracellular calcium phosphate in osteoblast-mediated bone apatite formation. Proc. Natl. Acad. Sci. USA.

[10] Nudelman F., Pieterse K., George A., Bomans P.H., Friedrich H., Brylka L.J., Hilbers P.A., de With G., Sommerdijk N.A. (2010). The role of collagen in bone apatite formation in the presence of hydroxyapatite nucleation inhibitors. Nat. Mater..

[11] Mahamid J., Aichmayer B., Shimoni E., Ziblat R., Li C., Siegel S., Paris O., Fratzl P., Weiner S., Addadi L. (2010). Mapping amorphous calcium phosphate transformation into crystalline mineral from the cell to the bone in zebrafish fin rays. Proc. Natl. Acad. Sci. USA.

[12] Du C., Cui F.Z., Zhang W., Feng Q.L., Zhu X.D., de Groot K. (2000). Formation of calcium phosphate/collagen composites through mineralization of collagen matrix. J. Biomed. Mater. Res..

[13] Vranceanu M.D., Saban R., Albu M.G., Antoniac I. (2012). Preparation and characterization of collagen: Amorphous calcium phosphate composites. Revista de Pielarie Incaltaminte Leather Footwear J..

[14] Nathanael A.J., Oyane A., Nakamura M., Sakamaki I., Nishida E., Kanemoto Y., Miyaji H. (2017). In vitro and in vivo analysis of mineralized collagen-based sponges prepared by a plasma- and precursor-assisted biomimetic process. ACS Appl. Mater. Interfaces.

[15] Santhakumar S., Oyane A., Nakamura M., Koga K., Miyata S., Muratsubaki K., Miyaji H. (2020). In situ precipitation of amorphous calcium phosphate nanoparticles within 3D porous collagen sponges for bone tissue engineering. Mater. Sci. Eng. C.

[16] Kanemoto Y. (2020). Assessment of Bone Forming Ability of Apatite-Coated Collagen Scaffold Prepared by a Precursor-Assisted Biomimetic Process. Ph.D. Thesis.

[17] Kokubo T., Takadama H. (2006). How useful is SBF in predicting in vivo bone bioactivity?. Biomaterials.

[18] Kobayashi K., Anada T., Handa T., Kanda N., Yoshinari M., Takahashi T., Suzuki O. (2014). Osteoconductive property of a mechanical mixture of octacalcium phosphate and amorphous calcium phosphate. ACS Appl. Mater. Interfaces.

[19] Niu X., Liu Z., Tian F., Chen S., Lei L., Jiang T., Feng Q., Fan Y. (2017). Sustained delivery of calcium and orthophosphate ions from amorphous calcium phosphate and poly(L-lactic acid)-based electrospinning nanofibrous scaffold. Sci. Rep..

[20] Hu Q., Tan Z., Liu Y., Tao J., Cai Y., Zhang M., Pan H., Xua X., Tang R. (2007). Effect of crystallinity of calcium phosphate nanoparticles on adhesion, proliferation, and differentiation of bone marrow mesenchymal stem cells. J. Mater. Chem..

[21] Clèries L., Fernández-Pradas J.M., Morenza J.L. (2000). Bone growth on and resorption of calcium phosphate coatings obtained by pulsed laser deposition. J. Biomed. Mater. Res..

[22] Walsh W.R., Morberg P., Yu Y., Yang J.L., Haggard W., Sheath P.C., Svehla M., Bruce W.J.M. (2003). Response of a calcium sulfate bone graft substitute in a confined cancellous defect. Clin. Orthop. Relat. Res..

[23] Frayssinet P., Gineste L., Conte P., Fages J., Rouquet N. (1998). Short-term implantation effects of a DCPD-based calcium phosphate cement. Biomaterials.

[24] Bohner M., Lemaitre J. (2009). Can bioactivity be tested in vitro with SBF solution?. Biomaterials.

[25] Woo Y.C., Park S.S., Subieta A.R., Brennan T.J. (2004). Changes in tissue pH and temperature after incision indicate acidosis may contribute to postoperative pain. Anesthesiology.

[26] Barrère F., van Blitterswijk C.A., de Groot K. (2006). Bone regeneration: Molecular and cellular interactions with calcium phosphate ceramics. Int. J. Nanomed..

[27] Dorozhkin S.V. (2010). Amorphous calcium (ortho)phosphates. Acta Biomater..

[28] Burke E.M., Lucas L.C. (1998). Dissolution kinetics of calcium phosphate coatings. Implant Dent..

[29] Combes C., Rey C. (2010). Amorphous calcium phosphates: Synthesis, properties and uses in biomaterials. Acta Biomater..

[30] Fujii T., Ueno T., Kagawa T., Sakata Y., Sugahara T. (2006). Comparison of bone formation ingrafted periosteum harvested from tibia and calvaria. Microsc. Res. Technol..

[31] Lim J., Lee J., Yun H.S., Shin H.I., Park E.K. (2013). Comparison of bone regeneration rate in flat and long bone defects: Calvarial and tibial bone. Tissue Eng. Regen. Med..

